# Increased odds of patient-reported success at 2 years after anterior cruciate ligament reconstruction in patients without cartilage lesions: a cohort study from the Swedish National Knee Ligament Register

**DOI:** 10.1007/s00167-017-4592-9

**Published:** 2017-06-07

**Authors:** Eric Hamrin Senorski, Eduard Alentorn-Geli, Volker Musahl, Freddie Fu, Ferid Krupic, Neel Desai, Olof Westin, Kristian Samuelsson

**Affiliations:** 1 Department of Health and Rehabilitation, Institute of Neuroscience and Physiology, The Sahlgrenska Academy, University of Gothenburg, Gothenburg, Sweden; 2 Fundación García-Cugat, Barcelona, Spain; 3 Artroscopia GC, SL, Barcelona, Spain; 4 Mutualidad Catalana de Futbolistas – Delegación Cataluña, Federación Española de Fútbol, Barcelona, Spain; 5 Department of Orthopedic Surgery, Mayo Clinic, Rochester, MN USA; 6 Department of Orthopedic Surgery, University of Pittsburgh, Pittsburgh, PA USA; 7 Department of Orthopedics, Institute of Clinical Sciences, The Sahlgrenska Academy, University of Gothenburg, 431 80 Mölndal, Gothenburg, Sweden; 8 Department of Orthopedics, Sahlgrenska University Hospital, Mölndal, Sweden

**Keywords:** Register, Anterior cruciate ligament, ACL, KOOS, Anatomic, Checklist, Patient-reported outcome, Meniscus, Cartilage

## Abstract

**Purpose:**

To investigate whether the surgical technique of single-bundle anterior cruciate ligament (ACL) reconstruction, the visualization of anatomic surgical factors and the presence or absence of concomitant injuries at primary ACL reconstruction are able to predict patient-reported success and failure. The hypothesis of this study was that anatomic single-bundle surgical procedures would be predictive of patient-reported success.

**Methods:**

This cohort study was based on data from the Swedish National Knee Ligament Register during the period of 1 January 2005 through 31 December 2014. Patients who underwent primary single-bundle ACL reconstruction with hamstring tendons were included. Details on surgical technique were collected using an online questionnaire comprising essential anatomic anterior cruciate ligament reconstruction scoring checklist items, defined as the utilization of accessory medial portal drilling, anatomic tunnel placement, the visualization of insertion sites and pertinent landmarks. A univariate logistic regression model adjusted for age and gender was used to determine predictors of patient-reported success and failure, i.e. 20th and 80th percentile, respectively, in the Knee injury and Osteoarthritis Outcome Score (KOOS), 2 years after ACL reconstruction.

**Results:**

In the 6889 included patients, the surgical technique used for single-bundle ACL reconstruction did not predict the predefined patient-reported success or patient-reported failure in the KOOS_4_. Patient-reported success was predicted by the absence of concomitant injury to the meniscus (OR = 0.81 [95% CI, 0.72–0.92], *p* = 0.001) and articular cartilage (OR = 0.70 [95% CI, 0.61–0.81], *p* < 0.001). Patient-reported failure was predicted by the presence of a concomitant injury to the articular cartilage (OR = 1.27 [95% CI, 1.11–1.44], *p* < 0.001).

**Conclusion:**

Surgical techniques used in primary single-bundle ACL reconstruction did not predict the KOOS 2 years after the reconstruction. However, the absence of concomitant injuries at index surgery predicted patient-reported success in the KOOS. The results provide further evidence that concomitant injuries at ACL reconstruction affect subjective knee function and a detailed knowledge of the treatment of these concomitant injuries is needed.

**Level of evidence:**

Retrospective cohort study, Level III.

## Introduction

Patient-reported outcome measurements (PROMs) are utilized to highlight the patient’s opinion of treatment outcome [23]. For instance, PROMs are commonly used after anterior cruciate ligament (ACL) reconstruction where the Knee injury and Osteoarthritis Outcome Score (KOOS) is one of the most frequently reported ones in the literature [10, 20]. In the KOOS, it has been suggested that a functional recovery for patients after ACL reconstruction can be defined as the lower threshold for the 95% CI of healthy 18- to 34-year-old males [2, 16]. Moreover, a treatment failure has been suggested to be a KOOS QoL score of <44 [9].

Previous studies including the KOOS have found that approximately 20–30% of patients after ACL reconstruction achieve a functional recovery or treatment failure, respectively [2, 9, 12]. One of these studies conducted by Barenius et al. [2] investigated patient-related and surgery-related factors to predict functional recovery and treatment failure in a cohort of patients with ACL reconstruction between 2005 and 2008. With regard to treatment outcome, the authors found that previous surgery on the menisci and a patellar graft were predictors of treatment failure and negative predictors of functional recovery after ACL reconstruction. In addition, a medial meniscus suture or resection at the time of reconstruction was predictive of treatment failure. Since this study was conducted, surgical procedures have evolved, where the use of anatomic reconstruction techniques has increased and has produced improved results in both biomechanical and clinical studies, compared with the older non-anatomic techniques [13, 26]. To evaluate anatomic ACL reconstructions, a tool, the anatomic anterior cruciate ligament reconstruction scoring checklist (AARSC), has recently been published [5, 25]. With new opportunities to perform and evaluate ACL reconstructive surgery, it remains to investigate whether detailed knowledge of the surgical procedures, with special emphasis on anatomic reconstruction, is able to predict patient-related outcome after ACL reconstruction.

The purpose of this study was therefore to investigate whether a detailed knowledge of surgical procedures was able to predict which patients have good and poor subjective knee function 2 years after ACL surgery in the Swedish National Knee Ligament Register (SNKLR). Specifically, the aim was to investigate whether the surgical technique of single-bundle ACL reconstruction, the visualization of anatomic surgical factors and the presence or absence of concomitant injuries at primary ACL reconstruction were able to predict patient-reported success and failure. The hypothesis of this study was that anatomic single-bundle surgical procedures would be predictive of patient-reported success. Increased knowledge of which patients do well or worse after treatment may in the future potentially help in terms of selecting appropriate for each individual patient.

## Materials and methods

Patient data were extracted from the SNKLR. Inclusion comprised patients who were registered for primary ACL reconstruction from 1 January 2005 to 31 December 2014. Patients aged 13–49 years who underwent single-bundle ACL reconstruction with hamstring autografts were eligible for inclusion. Follow-up started on the date of the primary ACL reconstruction and ended at the 2-year follow-up, so patients with incomplete data in the KOOS at the 2-year follow-up were excluded. Patients who underwent contralateral ACL or revision ACL surgery before the 2-year follow-up were excluded. Patients were also excluded if the exact dates of index ACL reconstruction or revision surgery or the exact details of the surgeon who performed the surgery were missing. The inclusion and exclusion criteria are summarized in Table 1.

**Table 1:** Summary of inclusion and exclusion criteria

Inclusion criteria
Primary ACL reconstruction
ACL reconstruction using hamstring tendon autograft
Single-bundle ACL reconstruction
Exclusion criteria
Non-primary ACL reconstruction
Non-ACL reconstruction
Year of surgery after 2013
Age other than 13–49 years
Graft type other than hamstring tendon autograft
Concomitant ligament injury requiring repair/reconstruction
Concomitant fracture/tendon injury
Concomitant vascular injury
Early contralateral ACL or revision surgery, within 550 days of index surgery
Incomplete data in the KOOS at the 2-year follow-up

*ACL* anterior cruciate ligament, *KOOS* Knee injury and Osteoarthritis Outcome Score

### The Swedish National Knee Ligament Register

The SNKLR is a nationwide database that collects prospective data on ACL injuries and associated knee surgery. The register utilizes a web-based protocol consisting of two parts: one surgeon-reported section and one patient-reported section. The surgeon-reported section includes information on the patients’ activity at the time of injury, time from injury to reconstruction, graft selection, fixation techniques and previous surgery. The surgeon registers all surgical procedures on the injured knee, including concomitant injuries and treatment of the meniscus and cartilage. The patient-reported section includes two PROMs, the KOOS [20] and *European Quality of Life*-*5 Dimensions* (EQ-5D) [17] for health-related quality of life. The SNKLR has reported a coverage (proportion of participating units in relation to all eligible units) of 92.9% and completeness (proportion of target population in the register) of >90%, with a 50–70% response rate for the patient-reported outcome measurements [8]. Additionally, a non-response analysis has been done showing that the register is valid despite the suboptimal number of patients responding at follow-up [18]. The register complies with the Swedish legislation relating to data security. All extracted data are anonymous, and investigators only have access to unidentifiable patient data. Participation in the SNKLR is voluntary for patients and surgeons. No written consent is necessary for national registers in Sweden.

### Surgical technique of single-bundle ACL reconstruction

To evaluate the surgical technique, single-bundle ACL reconstruction, an online questionnaire was created to collect detailed information from ACL surgeons in Sweden. The questionnaire included items from the anatomic anterior cruciate ligament reconstruction scoring checklist (AARSC) [5]. The AARSC has been tested for validity and reliability [25]. The questionnaire consists of 17 items covering the surgical technique and one item relating to the documentation of bone tunnel placement. The checklist allows for the calculation of an “anatomic score” with a total of 19 points [4, 5]. A total of 108 (61.7%) surgeons completed the questionnaire, with a mean nationwide AARSC score based on the questionnaire answers of 13.84 points. The results allowed for a time interval in which the single-bundle surgical techniques that were used could be identified for each surgeon who responded. Accordingly, through the SNKLR, the corresponding patients and surgical techniques could be determined.

### Groups

Groups were created with specific combinations of single-bundle surgical techniques based on eight relevant items selected from the questionnaire. Each group had a mandatory “Yes” or “No” answer requirement for certain items that subsequently identified that particular group (Table 2).

**Table 2:** Answer requirements characterizing defined groups^a^

	Use of an acc. medial portal	Visualization of the femoral ACL insertion site	Visualization of the tibial ACL insertion site	Lateral intercondylar ridge identified	Bifurcate ridge identified	Placing the femoral tunnel(s) in the femoral ACL insertion site	Placing the tibial tunnel(s) in the tibial ACL insertion site	Transportal drilling of the femoral ACL tunnel(s)
Group								
TP reference	Yes	Yes	Yes	Yes	Yes	Yes	Yes	Yes
TP anatomic						Yes	Yes	Yes
TT anatomic						Yes	Yes	No
TT partial-anatomic						No	Yes	No
TT non-anatomic						No	No	No
All landmarks		Yes	Yes	Yes	Yes			
No landmarks		No	No	No	No			
TP drilling								Yes
TT drilling								No

*Acc* Accessory, *TP* Transportal, *TT* Transtibial

^a^ Empty spaces are not assigned a mandatory answer requirement. Surgeons can thus answer “Yes” or “No” to these items

### Outcome

The main outcome and dependent variable was the KOOS_4_. The KOOS_4_ is an average score of four KOOS subscales, in which “function in daily living” is excluded to avoid a ceiling effect [9], because relatively young and active patients, like the patients in this study, rarely have difficulties with function in daily living. We studied the KOOS_4_ as a predictor in two ways: patient-reported success, defined as ≥80th percentile, and patient-reported failure, defined as ≤20th percentile. The definitions were chosen to highlight good and poor subjective outcomes after ACL reconstruction reflected by the top and bottom quantiles in the SNKLR. Surgical techniques of single-bundle ACL reconstruction, surgical factors and concomitant injuries were set as independent variables. The Regional Ethical Review Board in Gothenburg, Sweden, approved this study (D-nr: 760-14).

### Statistical analysis

Tables were generated using Microsoft Word (Version 14.0.7, Microsoft Corp, Redmond, Washington, USA). A statistician assigned to the SNKLR performed all the statistical analyses. Statistical analysis was performed in IBM SPSS Statistics (Version 23.0, IBM Corp, Armonk, New York, USA). Data were characterized according to the level of measurement as nominal scale data and ratio scale data. The means of normally distributed continuous data were compared with the independent-samples *t* test. Univariate logistic regression was used to predict patient-reported success and failure and is reported as an odds ratio (OR) with a 95% confidence interval. The univariate logistic regression model was adjusted for significant differences in patient baseline characteristics; age and gender. Statistical significance was determined at an alpha level of 0.05.

## Results

**Fig. 1:**
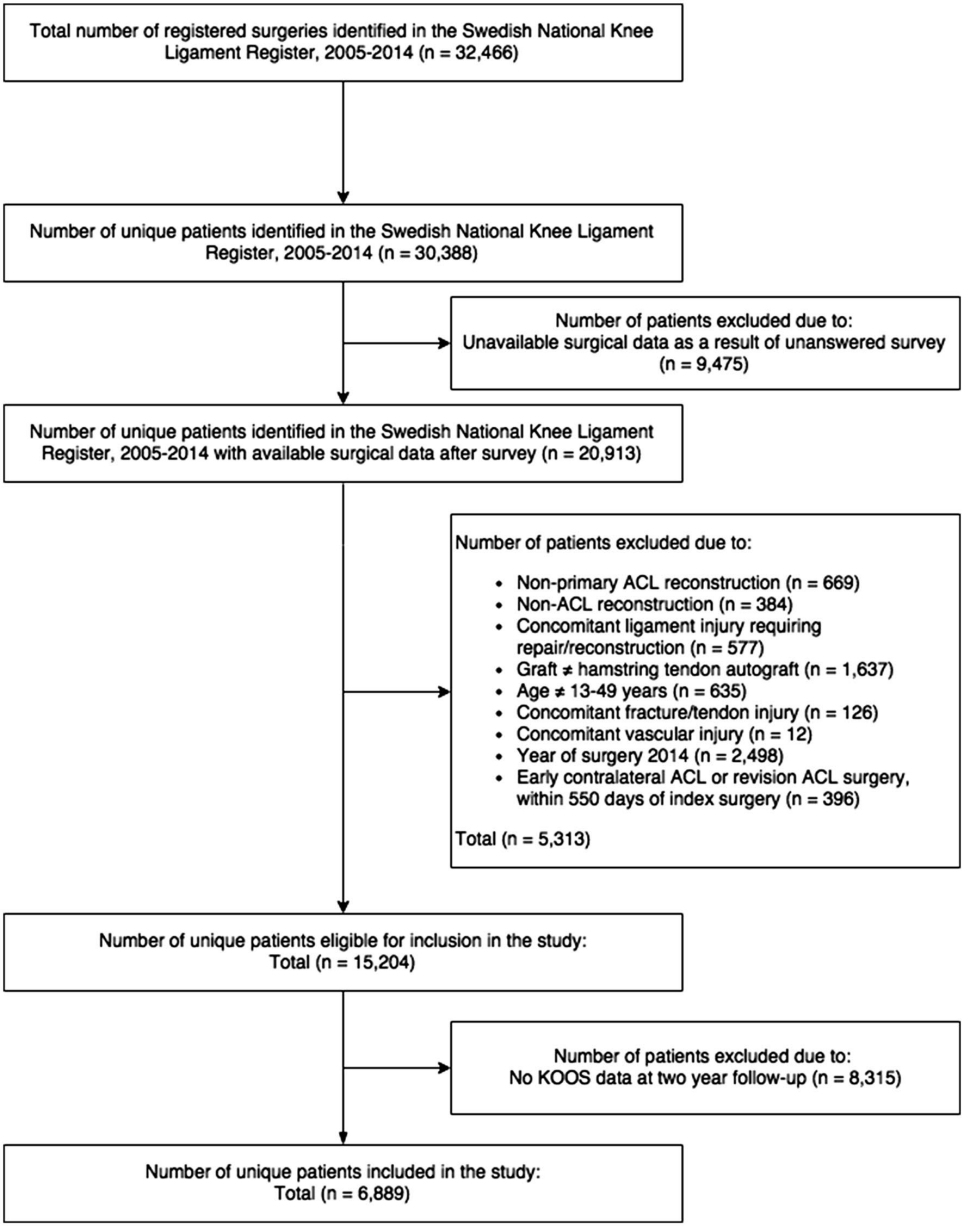
Flow chart demonstrating the selection of eligible patients from the Swedish National Knee Ligament Register

Data from 30,388 unique patients identified in the SNKLR between January 2005 and December 2014. Of these patients, a total of 20,913 were eligible for inclusion and, after applying all the exclusion criteria, 6889 patients were included in the study, 3461 females (50.2%) and 3428 males (49.8%) (Fig. 1). Males had a higher subjective knee function measured with the KOOS_4_ for the age of 7–25 years (*p* < 0.001) and 31–40 years (*p* < 0.01). On average, the 20th and 80th percentiles of the KOOS_4_ were higher for males (*p* < 0.001) (Table 3).

**Table 3:** Average subjective knee function scores at 2 years after ACL reconstruction

	Total cohort (*n* = 6889)							
	Female				Male			
Age at index ACL reconstruction	*N* (% of cohort)	KOOS_4_ at 2 year follow-up (Mean + SD)	20th percentile of KOOS_4_ (Mean)	80th percentile of KOOS_4_ (Mean)	*N* (% of cohort)	KOOS_4_ at 2 year follow-up (Mean + SD)	20th percentile of KOOS_4_ (Mean)	80th percentile of KOOS_4_ (Mean)
Total	3461 (50)	71.1 ± 19.2	53.9	88.4	3428 (50)	74.3 ± 18.8	58.4	91.3
13–15	415 (72)	73.2 ± 18.0	55.9	90.5	165 (28)	80.8 ± 16.1	69.7	94.9
16–20	1259 (65)	69.2 ± 18.7	52.2	86.4	676 (35)	72.7 ± 18.6	55.3	89.9
21–25	553 (45)	68.9 ± 19.1	50.9	86.5	675 (55)	71.2 ± 19.2	54.2	89.3
26–30	323 (34)	76.3 ± 15.6	61.5	90.9	633 (66)	74.7 ± 18.8	57.7	90.5
31–35	241 (34)	71.4 ± 21.2	49.8	91.3	470 (66)	75.5 ± 18.4	60.5	91.6
36–40	276 (41)	71.0 ± 21.1	53.7	88.5	403 (59)	76.2 ± 18.6	62.1	92.8
40–49	394 (49)	73.9 ± 19.6	57.1	90.4	406 (51)	75.7 ± 19.1	59.6	93.6

*ACL* anterior cruciate ligament, *KOOS* Knee injury and Osteoarthritis Score, *SD* standard

### Patient-reported success

Patient baseline characteristics for patient-reported success, i.e. 80th percentile or higher, and the cohort below the 80th percentile in the KOOS_4_ are presented in Table 4. No differences in the proportion of surgical techniques or surgical procedures were found. The lowest proportion of patients represented in the 80th percentile had undergone ACL reconstruction using the transtibial (TT) partial-anatomic technique. TT partial-anatomic was therefore used as a reference in the logistic regression model.

**Table 4:** Baseline characteristics of patients stratified by patient-reported success, i.e. ≥80th percentile

	Success ≥80th percentile		<80th percentile	
	*N*	%	*N*	%
Concomitant injury (*n* = 1373 vs 5516)				
MCL	31	17.4	147	82.6
LCL	10	24.4	31	75.6
Meniscus	529	18.1	2400	81.9
Cartilage	297	16.0	1558	84.0
Surgical techniques (*n* = 1189 vs 4797)				
TP reference (*n* = 2256)	442	19.6	1814	80.4
TT non-anatomic (*n* = 581)	117	20.1	464	79.9
TT anatomic (*n* = 944)	208	22.0	736	78.0
TT partial-anatomic (*n* = 702)	120	17.1	582	82.9
TP anatomic (*n* = 1503)	302	20.1	1201	79.9
Surgical factors				
Landmarks (*n* = 3789)	76	19.4	315	80.6
Footprints (*n* = 5951)	106	18.0	484	82.0
Ridges (*n* = 5231)	285	20.8	1083	79.2
Drilling TP versus TT (*n* = 6831)	446	19.6	1832	79.9

*MCL* medial collateral ligament, *LCL* lateral collateral ligament, *TP* transportal, *TT* transtibial

The absence of a concomitant injury to the menisci was significantly associated with patient-reported success (odds ratio [OR] = 0.81 [95% CI, 0.72–0.92], *p* = 0.001), as was the absence of cartilage injury (OR = 0.70 [0.61–0.81], *p* < 0.001). No associations with surgical techniques or surgical factors were found. However, a subassociation was found in favour of the TT partial-anatomic over the TT anatomic surgical technique (OR = 1.37 [95% CI, 1.07–1.76], *p* = 0.013) (Table 5).

**Table 5:** Logistic regression model for prediction of patient-reported success adjusted for age and gender, i.e. ≥80th percentile

Predictor	Odds Ratio	95% CI	*p* value
Concomitant injury			
MCL	0.84	0.57–1.25	n.s.
LCL	1.30	0.64–2.65	n.s.
Meniscus	0.81	0.72–0.92	0.001
Cartilage	0.70	0.61–0.81	<0.001
*Surgical techniques*			n.s.
Reference = TT partial-anatomic			
TP reference	1.18	0.95–1.48	n.s.
TT non-anatomic	1.22	0.92–1.62	n.s.
TT anatomic	1.37	1.07–1.76	0.013
TP anatomic	1.22	0.97–1.54	n.s.
Surgical factors			
Landmarks	1.06	0.81–1.38	n.s.
Footprints	1.17	0.94–1.46	n.s.
Ridges	0.95	0.81–1.10	n.s.
Drilling (TT vs TP)	1.04	0.91–1.17	n.s.

Statistical Significance *p* < 0.05

*MCL* medial collateral ligament, *LCL* lateral collateral ligament, *TT* transtibial, *CI* Confidence Interval

### Patient-reported failure

Patient baseline characteristics for patient-reported failure, i.e. 20th percentile or below, and the cohort above the 20th percentile in the KOOS_4_ are presented in Table 6. No differences in the proportion of surgical techniques or surgical procedures were found. The lowest proportion of patients represented in the 20th percentile had undergone ACLR using the TT non-anatomic technique. TT non-anatomic was therefore used as a reference in the logistic regression model.

**Table 6:** Baseline characteristics of patients stratified for patient-reported failure, i.e. ≤20th percentile

	Failure ≤20th percentile		>20th percentile	
	*N*	%	*N*	%
Concomitant injury (*n* = 1373 vs 5516)				
MCL	38	21.3	140	78.7
LCL	13	31.7	28	68.3
Meniscus	610	20.8	2319	79.2
Cartilage	423	22.8	1432	77.2
Surgical techniques (*n* = 1193 vs 4793)				
TP reference (*n* = 2256)	471	20.9	1785	79.1
TT non-anatomic (*n* = 581)	99	17.0	482	83.0
TT anatomic (*n* = 944)	172	18.2	772	81.8
TT partial-anatomic (*n* = 702)	152	21.7	550	78.3
TP anatomic (*n* = 1503)	299	19.9	1204	80.1
Surgical factors				
Landmarks (*n* = 3789)	79	20.2	312	79.8
Footprints (*n* = 5951)	121	20.5	469	79.5
Ridges (*n* = 5231)	257	18.8	1111	81.2
Drilling TP versus TT (*n* = 6831)	440	19.3	1838	79.7

*MCL* medial collateral ligament, *LCL* lateral collateral ligament, *TP* transportal, *TT* transtibial

The presence of a concomitant cartilage injury was significantly associated with patient-reported failure (OR = 1.27 [95% CI, 1.11–1.44], *p* = 0.001). No associations with surgical techniques or surgical factors were found. However, a subassociation was found in favour of the TT non-anatomic technique compared with the transportal (TP) reference surgical technique (OR = 1.37 [95% CI, 1.07–1.76], *p* = 0.013) (Table 7).

**Table 7:** Logistic regression model for prediction of patient-reported failure adjusted for age and gender, i.e. ≤ 20th percentile

Predictor	Odds ratio	95% CI	*p* value
Concomitant injury			
MCL	1.09	0.76–1.57	n.s.
LCL	1.87	0.97–3.62	n.s.
Meniscus	1.1	0.98–1.24	n.s.
Cartilage	1.27	1.11–1.44	<0.001
*Surgical techniques* Reference = TT non-anatomic			n.s.
TP reference	1.29	1.01–1.63	0.04
TT anatomic	1.09	0.83–1.42	n.s.
TT partial-anatomic	1.35	1.02–1.78	0.04
TP anatomic	1.21	0.94–1.55	n.s.
Surgical factors			
Landmarks	1.00	0.77–1.30	n.s.
Footprints	0.98	0.79–1.21	n.s.
Ridges	1.08	0.92–1.26	n.s.
Drilling (TT vs TP)	1.06	0.94–1.20	n.s.

Statistical Significance *p* < 0.05

*MCL* medial collateral ligament, *LCL* lateral collateral ligament, *TT* transtibial, *TP* transportal, *CI* Confidence Interval

## Discussion

The main finding was that the absence of concomitant injury to the meniscus and articular cartilage predicted patient-reported success. In addition, the presence of a concomitant injury to cartilage was a predictor of patient-reported failure. Moreover, the surgical technique in single-bundle ACL reconstruction did not predict patient-reported success or failure in the KOOS_4_ at 2 years.

Injuries to the ACL are common and a reconstruction is one of the most commonly performed outpatient orthopaedic surgeries. Although there is general agreement that it is important to provide axial and rotational stability in the course of surgical reconstruction, the optimal method for doing so remains controversial. It has been suggested that non-anatomically placed grafts are exposed to fewer forces compared with anatomically placed grafts [13]. The non-anatomically placed grafts may also result in residual rotational laxity of the knee, creating persisting instability [6]. This instability may cause the patient to adapt his/her behaviour and activity level, which could potentially affect subjective knee function. Nevertheless, it has been suggested that PROMs provide an indirect measurement of functional stability [24], but the present results may imply that the KOOS is suboptimal and is unable to detect any difference in knee kinematics affected by surgical technique in the short term. Additionally, the KOOS does not include any subscale or question related to perceived instability and the outcome could be too coarse to detect surgery-related differences in the knee. It is also possible to question whether the items in the KOOS are at all relevant when it comes to evaluating surgical outcome after ACL reconstruction. On the other hand, objective measurements of knee stability, such as a quantifiable pivot shift test, may be more appropriate for evaluating surgical technique and identifying small differences in knee-joint kinematics after using different ACL reconstruction techniques. However, no data on objective measurements of knee stability are kept at follow-up in the SNKLR.

Single-surgical factors, such as the identification of landmarks, footprints and both ridges, in addition to transtibial or transportal drilling, did not predict patient-reported outcome 2 years after ACL reconstruction in this cohort. Similarly, with regard to single-surgical factors, Duffee et al. [7] compared the transtibial and transportal drilling techniques and reported no association between femoral tunnel drilling and KOOS Sport and KOOS Quality of Life. However, the authors reported that patients who underwent ACL reconstruction where a transtibial technique was used to drill the femoral tunnel had significantly higher odds of undergoing repeat ipsilateral knee surgery compared with those in whom the femoral tunnel had been drilled using an anteromedial portal technique. The authors dichotomized repeat ipsilateral knee surgery after primary ACL reconstruction as “Yes” or “No”, including revision ACL surgery, meniscus and cartilage treatment.

In this study, no analysis of concomitant injuries with regard to drilling technique was performed. However, it was shown that an articular cartilage injury at ACL reconstruction significantly affected patient-reported success and failure 2 years after reconstruction. The absence of an articular cartilage injury was a predictor of patient-reported success, but, in comparison, the presence of an articular cartilage injury was a predictor of patient-reported failure. It is possible to question whether the dichotomization of concomitant injuries, such as articular cartilage injury “Yes” or “No” in our study, may be insufficient when it comes to truly predicting patient-reported outcome after ACL reconstruction. Additionally, the treatment of cartilage or meniscus injury was not controlled for in this study. It might therefore might be possible that these injuries predicted a patient-reported failure only because they were not treated, or adequately treated, and not by the presence of the injury itself. Partly confirming this, Cox et al. [3] showed that grade 3 and 4 articular cartilage lesions in various regions at index ACL reconstruction predicted poorer subjective knee function in the KOOS and International Knee Documentation Committee 6 years after surgery. Nevertheless, a concomitant injury to the articular cartilage appears to have a negative effect on patient-reported outcome [3, 14, 21]. This is a concern in terms of future degenerative changes, osteoarthritis and long-term outcome among these patients [15, 19]. In the future, it is recommended that the emphasis should be placed on treatment strategies aimed at restoring biomechanical function and delaying degenerative changes. Cox et al. [3] also found that medial meniscus injury and treatment at ACL reconstruction negatively affected patient-reported outcome 2 years after surgery. In our cohort, the absence of a meniscus was predictive of patient-reported success, but no effect was found for patient-reported failure. Interestingly, Barenius et al. [2], who investigated functional recovery and treatment failure after ACL reconstruction, found no effect of meniscus injury alone at the time of reconstruction in the KOOS 2 years after surgery. However, in their cohort, also from the SNKLR, a medial meniscus injury that required surgery was a predictor of treatment failure. Taken together, the results appear to provide further evidence of the improved patient-reported outcome in the short term when the meniscus is preserved at ACL reconstruction [2, 3, 21, 22].

The most distinctive potential limitation of this study was the incomplete response to the questionnaire sent out to the surgeons and any recall bias. Nevertheless, the retrospective collection of detailed surgical data was necessary to obtain information relating to items in the AARCS. At present, the data kept in the SNKLR alone are insufficient to evaluate anatomic ACL reconstruction. Assuming correct answers from the questionnaire, the surgeons can still erroneously recall dates at which a certain technique was adopted. To minimize recall bias, responders were asked only to answer the questions if they were sure of the date, by specifying specific years and not months, on which they adopted or abandoned the surgical technique in question. Moreover, all patients who underwent surgery during time periods when the surgeon was “in between” surgical techniques were not included [5]. There were also a large number of patients in the SNKLR with incomplete data and which therefore were excluded from the study. A non-response analysis of the SNKLR has been done showing that the register is valid despite the suboptimal number of patients responding at follow-up [18]; however, it cannot be ruled out that the incomplete data may have bias the results. Further limitations of the present study are that rehabilitation and pre-injury sports participation had not been controlled for. A higher pre-injury level of activity has been shown to increase the likelihood of treatment failure after ACL reconstruction [2]. In contrast, elite athletes have a higher rate of return to sport after surgery [1]. In addition, Grindem at el. [11] showed that patients who recover muscle strength and hop performance after ACL reconstruction are substantially less likely to sustain a re-injury to the ACL. Consequently, the incomplete data from muscle function and activity level may be confounders of our results. However, this is the first study to investigate whether anatomic single-bundle ACL reconstruction is able to predict patient-reported outcome 2 years after surgery. This study used the top and bottom quantiles of KOOS_4_ responses in the SNKLR, defined as patient-reported success and failure. Whether the corresponding KOOS_4_ for patient-reported success and failure correlates to the patients’ perception of treatment is not known. Recently, Ingelsrud et al. [12] investigated the proportion of patients who reported acceptable symptoms or treatment failure. Moreover, they also defined the corresponding KOOS values for each subscale of the patients’ perception of treatment outcome. The KOOS_4_ was not investigated, however, with regard to each subscale of the KOOS not including ADL, patients who reported acceptable symptoms had scores between 76 and 91 and patients who reported treatment failure had values between 31 and 58. The range presented for acceptable symptoms and treatment failure is extensive, but it does include the mean KOOS_4_ values for our cohort and may therefore by comparable. However, the possibility cannot be ruled out that the KOOS could be too coarse to enable the use of predefined percentiles to determine patient-reported success and failure.

The strengths of the study include the large sample size in which the data were gathered from the National Knee Ligament Register covering a whole country, which implies that the results are highly generalizable across different hospital settings. The study highlights the fact that, in clinical practice, PROMs such as the KOOS may be insufficient to evaluate the surgical techniques used in single-bundle ACL reconstruction. Additionally, the results provide further evidence that concomitant injuries to the articular cartilage and menisci at ACL reconstruction affect subjective knee function and a detailed knowledge of the treatment of these concomitant injuries with respect to the timing of ACL reconstruction is needed.

## Conclusion

In the present cohort study from the SNKLR, surgical technique was not predictive of patient-reported outcome in the KOOS_4_ 2 years after single-bundle ACL reconstruction. Patient-reported success was predicted by the absence of concomitant injury to the menisci and cartilage. The presence of a concomitant cartilage injury predicted patient-reported failure.
